# TMEM100 Regulates Neuropathic Pain by Reducing the Expression of Inflammatory Factors

**DOI:** 10.1155/2023/9151967

**Published:** 2023-07-10

**Authors:** Huifei Cui, Zhaoyang Guo, Zhu Guo, Zuoran Fan, Nana Shen, Xiaoying Qi, Yuanye Ma, Youfu Zhu, Xiaolin Wu, Bohua Chen, Hongfei Xiang

**Affiliations:** ^1^Department of Orthopedics, The Affiliated Hospital of Qingdao University, Qingdao 266003, China; ^2^Department of Orthopedics, The Second Affiliated Hospital of Chongqing Medical University, Chongqing 400010, China; ^3^Department of Rehabilitation, The Affiliated Hospital of Qingdao University, Qingdao 266000, China; ^4^Department of Gynecology, The Affiliated Hospital of Qingdao University, Qingdao 266003, China

## Abstract

There is no effective treatment for peripheral nerve injury-induced chronic neuropathic pain (NP), which profoundly impacts the quality of life of those affected. Transmembraneprotein100 (TMEM100) is considered to be a pain regulatory protein and is expressed in the dorsal root ganglion (DRG) of rats. However, the mechanism of pain regulation and the expression of TMEM100 following various peripheral nerve injuries are unclear. In this study, we constructed two pain models of peripheral nerve injury: tibial nerve injury (TNI) and chronic constriction injury (CCI). This study found that the Paw Withdrawal Mechanical Threshold (PWMT) and Paw Withdraw Thermal Latency (PWTL) of the rats in the two pain models decreased significantly, and the expression of TMEM100 in the DRG of two groups also decreased significantly. Furthermore, the decrease in the CCI group was more obvious than in the TNI group. There was no significant statistical significance (*P* > 0.05). We constructed an adeno-associated virus 6 (AAV6) vector expressing recombinant fluorescent TMEM100 protein and injected it into the sciatic nerve (SN) of two pain models: CCI and TNI. PWMT and PWTL were significantly increased in the two groups, along with the expression of TMEM100 in the spinal cord and DRG. It also significantly inhibited the activation of microglia, astrocytes, and several inflammatory mediators (TNF- *α*, IL-1 *β*, and IL-6). In summary, the results of this study suggested that TMEM100 might be a promising molecular strategy for the treatment of NP, and its anti-inflammatory effects might play an important role in pain relief.

## 1. Introduction

The term neuropathic pain (NP) refers to the pain caused by a primary lesion or dysfunction of the nervous system [1, 2]. It is a common clinical problem that usually manifests as persistent pain (burning, squeezing, and compression) or paroxysmal pain (shock-like sensations and tingling), resulting in paresthesia and dysesthesia (tingling and needles) [3]. NP affects approximately 7%–10% of the general population globally, primarily in patients over 50 [2, 4]. The pathogenesis of NP is complex. Previous studies have found that NP is associated with structural and functional changes in nociceptive pathways such as peripheral nerve injury sites, spinal cord, and dorsal root ganglia (DRG) [3]. These diseases are associated with various peripheral or central nervous system lesions [5]. NP can be divided into peripheral and central NP based on the anatomy of injury and disease [6]; the former is more common in clinical practice [7, 8]. Although peripheral nerve injury frequently causes chronic NP, the underlying mechanisms are often uncertain [9]. There are currently no adequate and effective treatments for NP, while numerous studies have focused on discovering new drug targets and their impact on pain behavior.

Transmembraneprotein100 (TMEM100) has been found to have many biological functions, expressed in vascular, lung, and gastrointestinal tissues, and plays an imperative role in arterial endothelial differentiation, vascular integrity, cancer cell, and proliferation [10–13]. Recently, it has been found that TMEM100 is also expressed in DRG [9, 14] as a pain signal modulator expressed by nociceptive neurons. It regulates pain by regulating the interaction between TRPA1 and TRPV1, and plays an important role in pain and the nervous system [14]. DRG is an important part of the peripheral nervous system. It is reported that TMEM100 is mainly located in mouse DRG and ganglion cell peptidergic neurons [14, 15], but its expression in NP is uncertain [15]. There may be differences in gene expression between inflammatory pain and NP [16–19].

Studies have shown that chronic pain is caused by interference with the decomposition of neuroinflammation [20–23]. Typical features of neuroinflammation under chronic pain conditions include infiltration of immune cells into the sciatic nerve (SN) and DRG, activation of glial cells: microglia and astrocytes, production and secretion of pro-inflammatory cytokines and chemokines (TNF, IL-1 *β*, IL-6, CCL2, and CXCL1) [20]. Previous studies have reported that TMEM100 has an inhibitory role in the secretion of inflammatory cytokines in liver inflammation [24]. Therefore, we investigated the contribution of TMEM100 in the inception of inflammation in nerve tissue.

AAV6 is now considered one of the most useful vectors for gene therapy due to its less immunogenicity and toxicity. Gene delivery to the DRG has been shown to be possible. It has been reported that retrograde transfection of AAV6 into neurons by SN injection can achieve higher transfection efficiency of DRG neurons than other transfection methods (intravenous, intramuscular, and intrathecal).

AAV6 is now considered one of the most useful vectors for gene therapy due to its less immunogenicity and toxicity [25]. Gene delivery to the DRG has been shown to be possible [26, 27]. It has been reported that retrograde transfection of AAV6 into neurons by SN injection can achieve higher transduction efficiency of DRG neurons than other transduction methods (intravenous, intramuscular, and intrathecal) [28, 29].

This study aimed to explore the effect of TMEM100 on NP and the therapeutic potential of TMEM100 as a target for treating chronic NP. In this study, we constructed an adeno-associated virus (AAV6) -TMEM100 overexpression vector and injected it into the SN of the rats, and analyzed the differential expression of TMEM100 and pain behavior in rats. In addition, the pro-inflammatory cytokines causing pain in rat DRG were evaluated.

## 2. Methods and Materials

### 2.1. Animals

This study used clean, healthy male Sprague–Dawley (SD) rats aged 7–10 weeks, weighing 200–300 g. The indoor temperature was maintained at 23 ± 2°C, the relative humidity was 60%–70%. The rats were fed in separate cages and drank and ate freely. The Qingdao University Experimental Animal Center in Shandong Province provided all the experimental animals. The experimental operation met the requirements of Animal Protection Association and the user Committee of Qingdao University and was consistent with the guidelines of National Animal Protection Institute.

### 2.2. Establishing Animal Models

According to the method of Bennett [30], a rat model of chronic constriction injury (CCI) was established: we used 4% isoflurane for induction and 2% for maintenance of anesthesia, then made the rat prone on the operating table, and the right hind limb was shaved for preparation. The skin was sterilized with 10% iodophor solution, followed by the incision at the sciatic tubercle and blunt dissection of each layer of muscle to expose the SN; 4–0 catgut was used to make four loose fit junctions, with a spacing of about 1 mm, to obtain a damaged SN of length 4–5 mm. The ligation strength was based on the mild compression of epineurial artery under the dissecting microscope, while the blood flow was not completely interrupted. When the rat exhibited typical NP signs: reduced hindlimb weight, paw contracture, licking, and no motor function limitation such as lameness, the model was judged to be successful.

According to the method of Lee et al. [31] a rat model of permanent tibial nerve injury (TNI) was established: the steps of anesthesia, skin preparation, and disinfection were the same as the CCI model. The SN and its three branches were exposed and separated from the surrounding soft tissue with a nerve dissection. The SN stem and three branches were carefully isolated. The tibial nerve branches were carefully identified, and the tibial nerve was tightly ligated with 4–0 chrome catgut and cut to a length of approximately 5 mm, leaving the common peroneal and sural nerves intact. The success sign was the same as CCI.

### 2.3. Experimental Grouping

A total of 80 male SD rats were included in this study and randomly divided into eight groups: normal; Sham; CCI; TNI; CCI, AAV6-GFP; TNI, AAV6-GFP; CCI, AAV6-TMEM100; and TNI, AAV6-TMEM100 group with 10 each. The description of groups is as follows.

Nothing was done to the normal group. In the Sham group, SN was exposed but not ligated. Surgical modeling was done in CCI and TNI groups, but no injection was administered. While in CCI, AAV6-GFP, and TNI, AAV6-GFP groups; AAV6-GFP (10^*∗*^ 1,012 viral particles each) were injected into the SN. Furthermore, in CCI, AAV6-TMEM100, and TNI, AAV6-TMEM100 groups, AAV6-TMEM100 (10^*∗*^ 1,012 viral particles each) were injected into the SN during modeling. AAV6-TMEM100 and AAV6-TMEM100 were designed and synthesized by Biomedicine Biotech (Chongqing, China).

### 2.4. Behavioral Analysis

Paw Withdrawal Mechanical Threshold (PWMT) test was conducted one day before modeling, and 1, 3, 5, 7, 10, 14, 21, and 28 days after modeling, according to the up–down method [32, 33], using von Frey filaments to determine mechanical allodynia by foot withdrawal. The rat was placed on a metal mesh frame, and the cilia were stabbed vertically through the mesh into the skin of rat's hind limbs until they were slightly bent into an S shape. The duration of each stimulation was 3–5 s, and the interval was 10–15 s. Reaction: if the rat displayed foot withdrawal, it was marked as “+”; if there was no response, it was marked as “−”; if “+” appeared, the adjacent cilia with decreasing force were used for stimulation; if negative, the adjacent increasing force was used for stimulation. Stimulation was stopped if there was no positive response to the maximum stimulation intensity of cilia. After the first positive reaction, the up–down method was used to repeat the measurement six times with a 10 min rest; the extreme values on both sides were removed, and the average of remaining values was taken as the PWMT value of rat.

Paw Withdraw Thermal Latency (PWTL) determination was done one day before modeling, and 1, 3, 5, 7, 10, 14, 21, and 28 days after modeling, according to the paw withdrawal latency (PWL) method [34]. The thermal radiation exposure time limit was set to 20 s, the rat was placed on the glass plate while the temperature of glass plate was maintained at 26 ± 0.5°C, and the irradiation light source under the glass plate was adjusted to aim at the palm of hind paw of the rat. When the foot withdrawal reaction occurred or the irradiation time reached 20 s or more (20 s was the irradiation limit), the light source was turned off and recorded. The measurement was repeated six times with a 10 min rest; the extreme values on both sides were removed, and the average was recorded as PWTL.

### 2.5. Immunofluorescence

After the pain behavior measurement, rats in each group were dissected at L4, L5, and L6 lumbar vertebrae and the DRG, and the DRG tissue was quickly removed and dissected. The L4–L6 spine was exposed, and the spinal cord was separated from the middle with a tool. The removed tissues were postfixed in 4% paraformaldehyde at 4°C for 8 hr, embedded in paraffin, and serially sectioned at 4 *µ*m. Immunofluorescence tristaining was done to characterize cellular specificity and distribution of target molecules in sections. Four micrometer thick sections were deparaffinized in xylene, rehydrated by graded alcohols, and treated by heat-induced epitope retrieval in 10 mM citrate buffer (Elabscience). We used a 5% BSA (Solarbio, Beijing, China) blocking solution for 1 hr at room temperature of 37°C, following the addition of primary antibody diluted with antibody diluent (dilution ratio was according to the antibody instructions) dropwise and the wet box was placed in a 4°C refrigerator to incubate overnight. Two fluorescent secondary antibodies (Elabscience) were added and incubated at room temperature at 37°C for 1 hr. After each incubation, wash the polyvinylidene fluoride (PVDF) membrane three times with TBST solution on a shaker for 10 min/time. Then, the nuclei were counterstained with the fluorescent dye DAPI (blue), and dropwise an antifluorescence quencher was added. Coverslips were placed in a low-temperature freezer at 4°C for later use. After the preparation, the slices were observed under a confocal microscope, and the tissue images were photographed using Image-Pro Plus (version 6.0.0.260, Media Cybernetics Corporation, USA). Images were finally analyzed.

### 2.6. Quantitative Real-Time Polymerase Chain Reaction (qRT–PCR)

After the removal of specimen, it was put into an enzyme-free EP tube (containing 1 ml Trizol (Elabscience) and steel ball) followed by shaker grinding at 60 rpm for 30 s, six times. After standing, chloroform was added to extract mRNA. It was then reverse transcribed into cDNA using the Evo M-MLV RT Kit for qPCR (Accurate Biology). The qPCR amplification reaction was performed on a PCR instrument according to the kit manufacturer's instructions (SYBR® Green Premix Pro Taq HS qPCR Kit (Accurate Biology)) with the following conditions: 95°C for 30 s, then 95°C for 5 s, and 60°C for 30 s for 40 cycles. The primer sequences used in this study are displayed in Table 1. The obtained data were analyzed using the 2(−*ΔΔ*Ct) algorithm to acquire the results.

### 2.7. Western Blot Analysis

Proteins were extracted by lysing tissues with radioimmunoprecipitation (RIPA) lysis buffer (Solarbio, Beijing, China) containing 1 mM phenylmethanesulfonyl fluoride (PMSF). The concentration of extracted protein was determined with a bicinchoninic acid (BCA) kit (Solarbio, Beijing, China). Protein and loading buffer were mixed at a ratio of 4 : 1 (V/V) and boiled at 99°C for 10 min. Separated by sodium dodecyl sulfate-polyacrylamide gel electrophoresis (SDS-PAGE) and transferred to PVDF membrane. PVDF membranes were blocked with 5% nonfat dry milk at room temperature. Membranes were then incubated with primary antibodies overnight at 4°C (Table 2). After overnight, the membrane was incubated with horseradish peroxidase (HRP)-labeled secondary antibody (Elabscience) for 1 hr, and then the ECL kit (Elabscience) was used for luminescence observation. The acquired images were analyzed using a developing instrument (Odyssey® XF).

### 2.8. Statistical Analysis

Statistical analysis was performed on the data collected, detected, and sorted. Continuous data were presented as mean ± standard deviation, while nonparametric data were presented as median and interquartile range. Comparisons between groups were performed using one-way analysis of variance (ANOVA) followed by the Kruskal–Wallis test. A comparison of parameters between parallel groups was performed using the *t*-test. *P* < 0.05 was considered statistically significant. Statistical analysis was performed, and statistical graphs were drawn using GraphPad Prism 8 (GraphPad Software, USA) software.

## 3. Results

### 3.1. Decreased TMEM100 Expression in the Two NP Models

#### 3.1.1. The Two Groups of Rat Pain Models Exhibited a Significant Decrease in Behavioral Pain Tests

**Figure 1:**
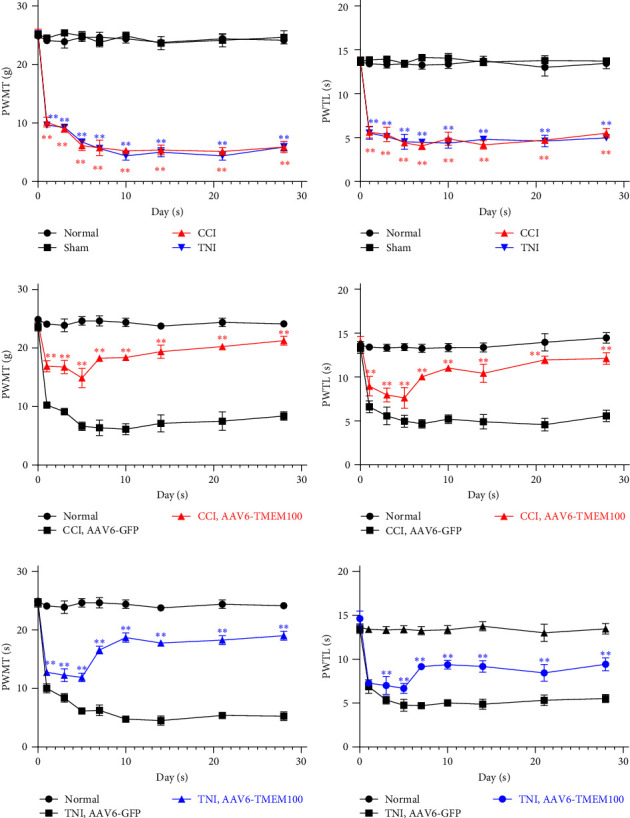
(a) Changes of PWMT in CCI and TNI groups at different times.  ^*∗∗*^*P* < 0.01 vs. normal group; (b) changes in PWTL in CCI and TNI groups. ^*∗∗*^*P* < 0.01 vs. normal group; (c) PWMT changes of rats in CCI group at different times after transfection; (d) changes of PWTL of rats in CCI group after transfection at different times; (e) rats in TNI group after transfection at different times; (f) changes in PWTL in TNI group rats at different times after transfection;  ^*∗∗*^*P* < 0.01 vs. AAV6-GFP group.

The postoperative condition of rats was good overall; and there was no autophagy of limbs, and typical spontaneous hyperalgesia gradually appeared. The right limb of rat was involved, dragged, or suspended, and there was obvious walking and lameness. We first conducted pain studies on the rats in the normal, Sham, CCI, and TNI groups: PWMT and PWTL test results revealed that the values of normal and Sham groups were the same each time, and there was no significant change (*P* > 0.05). Compared with the normal group, the PWMT and PWTL of the CCI and the TNI group decreased at each time point after the operation, and they decreased significantly on the first day and lasted until the 28th day after the operation (*P* < 0.01, Figures 1(a) and 1(b)).

#### 3.1.2. Painful Rats Transfected with Adenovirus Demonstrated Significant Relief in Pain Behavior

During modeling, we injected AAV6-GFP and AAV6-TMEM100 into the SN of CCI and TNI rats. The results indicated that the AAV6-TMEM100 group had a lesser decrease in PWMT and PWTL than the AAV6-GFP group, and in the last seven days after the operation, there were different degrees of increase (*P* < 0.01), with the highest level at 28 days (*P* < 0.01, Figure 1(c)–1(f).

#### 3.1.3. Decreased TMEM100 Expression of DRG in Peripheral NP

**Figure 2:**
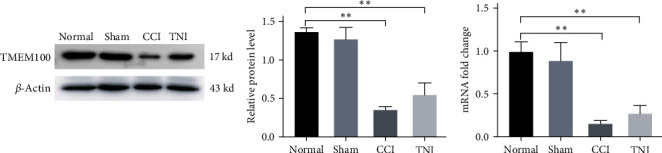
(a) Western blot used to detect the expression of TMEM100 protein in CCI and TNI groups; (b) qRT–PCR was used to detect the mRNA of TMEM100 in CCI and TNI groups.  ^*∗∗*^*P* < 0.01.

After 4 weeks of modeling, the rats were sacrificed, and their DRG tissues were dissected. Normal and Sham groups were compared using Western blot, qRT–PCR, and immunofluorescence. Moreover, DRG tissues of CCI and TNI groups were compared. The expression of TMEM100 gene varied, and the expression of TMEM100 in normal and Sham groups were detectable. The expression of TMEM100 was significantly attenuated in CCI and TNI groups compared to normal and Sham groups, with statistical significance (*P* < 0.01). The TNI group had slightly higher expression levels than the CCI group, but the difference was not statistically significant (*P* > 0.05, Figure 2(a)). The qRT–PCR results were consistent with western blot results. The expression in CCI and TNI groups was significantly decreased than in the normal and Sham groups (*P* < 0.01). CCI group histone expression was slightly lower than TNI (*P* > 0.05). Normal and Sham groups had similar histone expressions (*P* > 0.05, Figure 2(b)).

**Figure 3:**
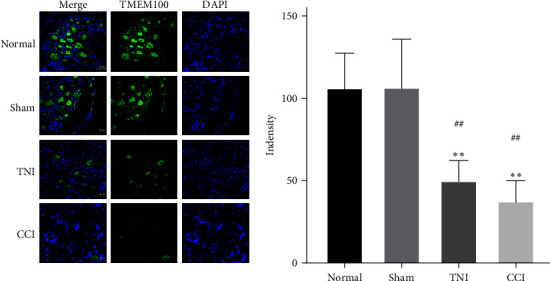
The relative fluorescence intensity of TMEM100 expression in DRG of CCI and TNI groups was detected by immunofluorescence.  ^*∗∗*^*P* < 0.01 vs. normal group; ^##^*P* < 0.01 vs. normal group.

Immunofluorescence staining yielded identical outcomes described previously. The fluorescence intensity of CCI and TNI groups was lower than normal and Sham groups (*P* < 0.05), TNI group was slightly higher than CCI group (*P* > 0.05), and there were no significant differences between the other groups (*P* > 0.05, Figure 3). By constructing two pain models, CCI and TNI, we discovered that TMEM100 expression was downregulated in the DRGs of both models. We speculated that TMEM100 might be involved in the occurrence of pain, as its expression exhibited a downward trend.

### 3.2. Minimally Invasive Injection of AAV6-TMEM100 into CCI and TNI Rats Could Reverse the Decrease in TMEM100 and Relieve NP

**Figure 4:**
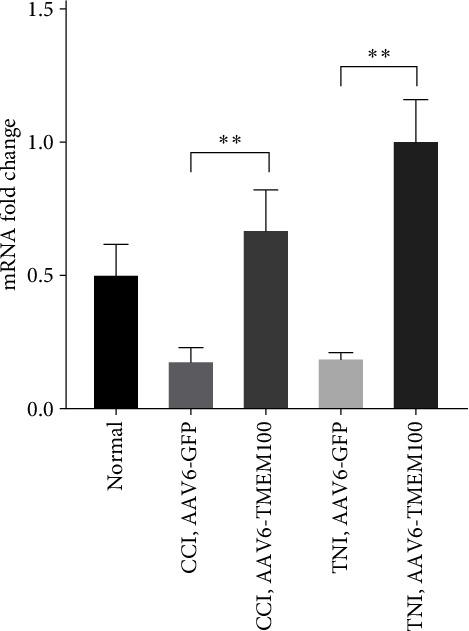
Relative expression of mRNA in normal group and virus transfection group  ^*∗∗*^*P* < 0.01.

Pain models: CCI and TNI were transfected with TMEM100 mediated by adenovirus, and the same experimental group was set up as a normal group and virus transfection group (AAV6-GFP and AAV6-TMEM100 groups). Four weeks after transfection, qRT–PCR detection was performed. The results revealed that AAV6-TMEM100 had significantly increased expression compared to AAV6-GFP group with a statistical difference (*P* < 0.05, Figure 4).

**Figure 5:**
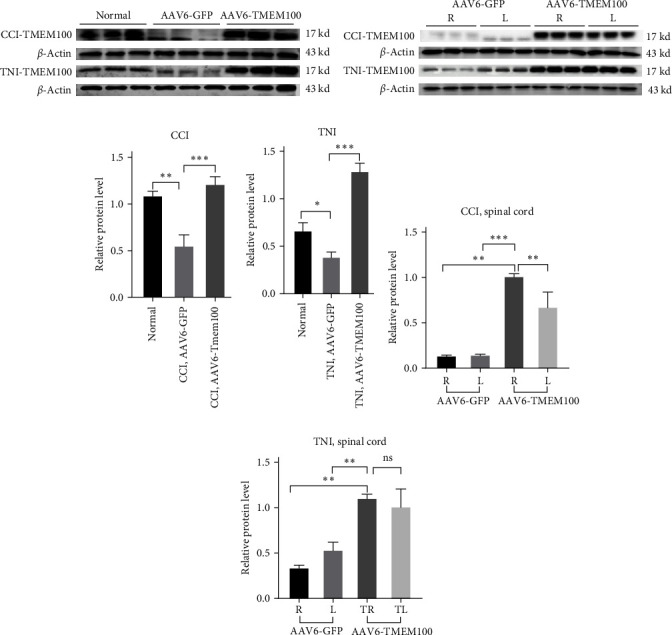
(a) The expression of TMEM100 protein in normal group and virus transfection group (AAV6-GFP and AAV6-TMEM100 groups). (b) The expression of CCI and TNI rat spinal cord in virus transfection group. The expression of TMEM100 protein on the model side (R) and the unmodeled side (L). (c) The histogram of expression of TMEM100 protein of CCI in the virus transfection group. (d) The expression of TMEM100 protein of TNI in the virus transfection group. The histogram of expression level. (e) The histogram of expression level of TMEM100 protein in the spinal cord of CCI rats in the virus transfection group. (f) The histogram of expression level of TMEM100 protein in the spinal cord of TNI rats in the virus transfection group;  ^*∗*^*P* < 0.05 and  ^*∗∗*^*P* < 0.01.

Western blotting detected that CCI, AAV6-TMEM100, and TNI, AAV6-TMEM100 groups had significantly enhanced protein expression compared with the AAV6-GFP group with statistical differences (*P* < 0.05, Figures 5(a), 5(c), and 5(d)). Simultaneously, we extracted and tested the spinal cords of rats' modeled side (*R*) and the unmodeled side (*L*) in each group, obtaining comparable results with the former. There was a statistical difference (*P* < 0.05). Furthermore, in CCI, the expression of TMEM100 in the modeled side of spinal cord of the rats in AAV6-TMEM100 group was higher than the unmodeled side, with a statistical difference between the two (*P* < 0.05). The expression of TMEM100 in the spinal cord of rats in the TNI and AAV6-TMEM100 groups was higher than on the nonmodeled side, but there was no significant difference between the two (*P* > 0.05, Figures 5(b), 5(e), and 5(f)).

**Figure 6:**
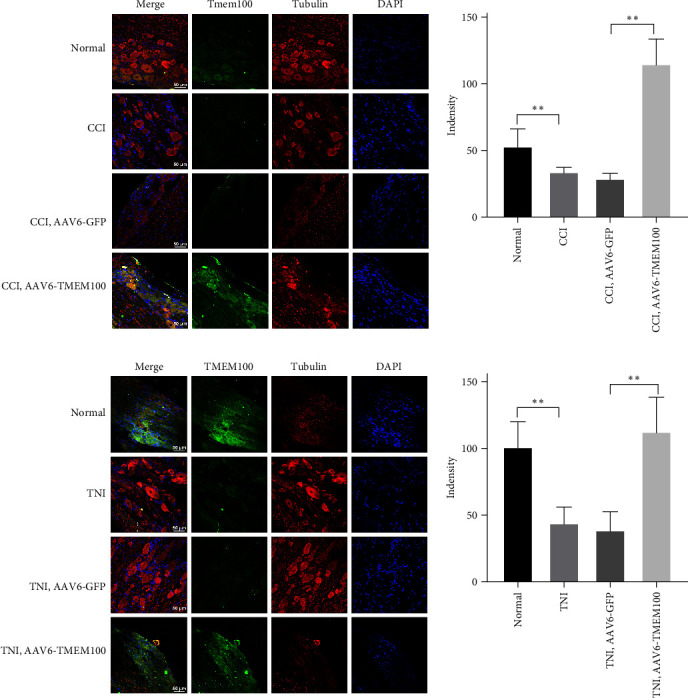
(a) Immunofluorescence detection of relative fluorescence intensity of TMEM100 in CCI rat normal group and virus transfection group (AAV6-GFP and AAV6-TMEM100 groups); (b) immunofluorescence detection of TNI rats. The relative fluorescence intensity expression difference of TMEM100 between normal group and virus transfection group (AAV6-GFP and AAV6-TMEM100 groups)  ^*∗∗*^*P* < 0.01.

Immunofluorescence staining yielded similar results as mentioned above. The AAV6-TMEM100 group exhibited a statistically significant enhanced fluorescence intensity compared to the AAV6-GFP group (*P* < 0.05, Figures 6(a) and 6(b)). This suggested TMEM100 as a crucial protein that regulates pain and immunity and plays a crucial role in NP. Transfection with adenovirus carrying TMEM100 could reverse discogenic pain and achieve the therapeutic effect. It could serve as gene therapy for discogenic pain. Sexual pain provides a good theoretical basis and a prerequisite for later animal experiments.

### 3.3. Reversal of TMEM100 Expression Reduces the Elevated Expression of Glial Cells and Inflammatory Mediators Caused by Peripheral NP

**Figure 7:**
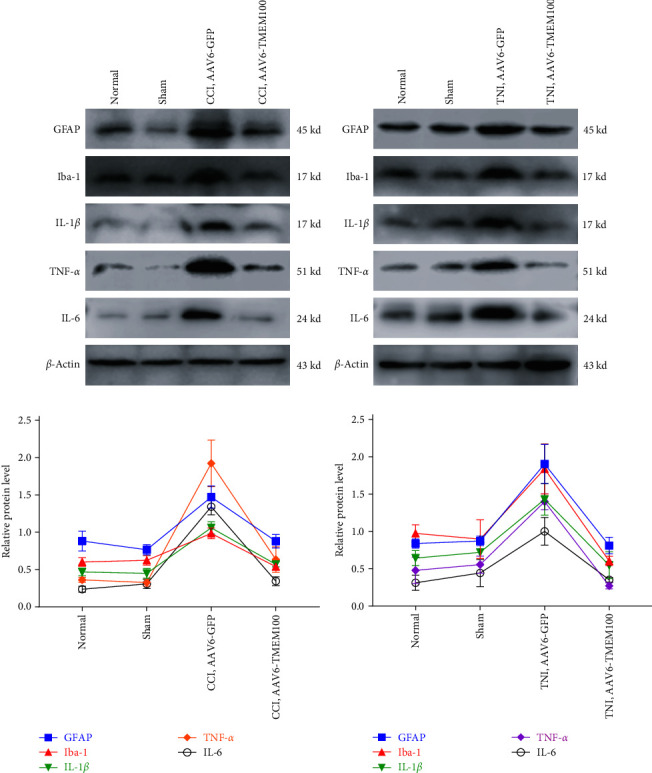
Western blot was used to detect the difference of protein expression of microglia (Iba-1), astrocytes (GFAP), and inflammatory mediators (TNF- *α*, IL-6, and IL-1 *β*) between CCI (a) and TNI (b) groups (AAV6-GFP group and AAV6-TMEM100 group). (c) Histogram of protein expression in CCI group; (d) histogram of protein expression in TNI group.

**Figure 8:**
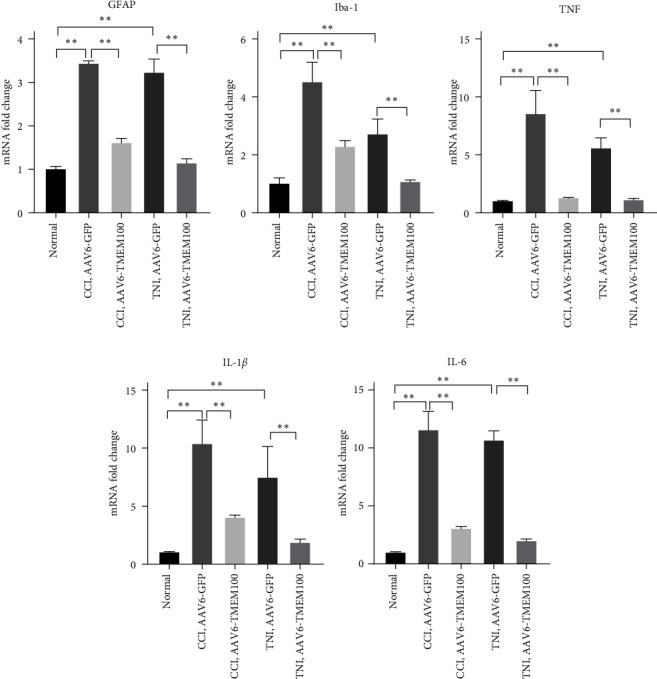
Detecting microglia (Iba-1), astrocytes (GFAP), and inflammatory mediators in virus-transfected groups (AAV6-GFP and AAV6-TMEM100 groups) in CCI and TNI groups by qRT–PCR detection (TNF-*α*, IL-6, and IL-1*β*) mRNA expression differences.  ^*∗∗*^*P* < 0.01.

This study investigated the relationship between TMEM100 and microglia (Iba-1), astrocytes (GFAP), and inflammatory mediators (TNF-*α*, IL-6, and IL-1*β*) after peripheral NP. Western blot, qPCR, and immunofluorescence were used to detect the expression levels of each index in the normal and the virus-injected groups. The qPCR demonstrated that the expressions of Iba-1, GFAP, TNF-*α*, IL-6, and IL-1*β* in the AAV6-GFP group were increased to varying degrees compared to the normal group (*P* < 0.05). The AAV6-TMEM100 group had significantly lower expression than the AAV6-GFP group (*P* < 0.05, Figure 7). Similar results were obtained by the western blot (*P* < 0.05, Figure 8).

**Figure 9:**
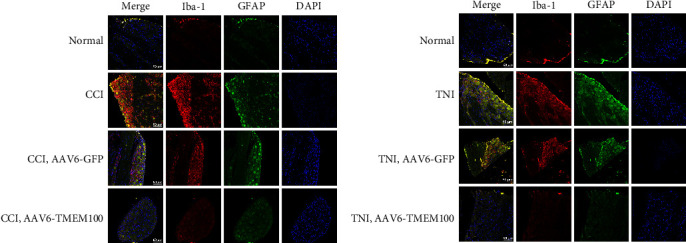
Detecting microglia (Iba-1), astrocytes (GFAP), and inflammatory mediators in virus-transfected groups (AAV6-GFP and AAV6-TMEM100 groups) in CCI and TNI groups by immunofluorescence detection (TNF-*α*, IL-6, and IL-1*β*) fluorescence intensity expression difference.

We further verified the expression of Iba-1 and GFAP by immunofluorescence, and the results were consistent with those obtained by qPCR and Western blot (Figure 9).

These results indicated that TMEM100 might relieve pain by reducing the expression of glial cells and inflammatory factors in NP.

## 4. Discussion

This study aimed to determine the expression of TMEM100 in NP and to explore the possible role of TMEM100 in pain relief. We found that the expression of TMEM100 was significantly reduced in the DRG of rats with peripheral NP by creating two different pain models: CCI and TNI. We established an AAV6 vector encoding recombinant fluorescent TMEM100 and transfected it into the DRG proximal to the peripheral nerve injury. We found that the expression of TMEM100 in the DRG of transfected rats was significantly higher than that of model rats alone, and the pain behavior of rats was significantly improved. Moreover, we discovered that reversing the expression of TMEM100 inhibited NP and microglia (Iba-1), astrocytes (GFAP), and inflammatory mediators (IL-1*β*, TNF-*α*, and IL-6). Overall, the findings of this study direct that TMEM100 is an important pain-regulating protein that plays an important role in NP and may alleviate pain by reducing inflammatory mediators.

TMEM100 is a two-transmembrane protein that is widely distributed in various tissues. It has been reported that TMEM100 is expressed in blood vessels, notochords, and other tissues and is related to kidney development, angiogenesis, and lung cancer metastasis [10, 35, 36]. The expression of TMEM100 has recently been found in the nervous system [14]. However, the expression and role of TMEM100 in NP are still unclear.

To further determine the association between TMEM100 and pain, we established two pathological pain models: CCI and TNI. The CCI model is a well-established [30] and the most used pain model in research. Pain is induced by the compression of four thread knots of the SN trunk, and rats may experience paresthesia, mechanical allodynia, and caloric allodynia in the operated limb, similar to the characteristics of human NP [37]. The TNI model is an optimized derivative type of spared nerve injury (SNI). It has the typical characteristics of SNI class and some advantages. Lee et al. [31] found that simultaneous transection of the tibial and sural nerves or a single TNI resulted in more severe pain threshold changes. The tibial nerve may play an important role in the pain process [38]. Therefore, researchers believed single TNI to be a more stable and efficient model of peripheral NP than classic SNI [39].

We performed behavioral tests on rats with two different pain models and evaluated the expression of TMEM100 in each group. We found that the pain production was accompanied by changes in the TMEM100 expression in the DRG of rats in the two pain models. The expression of TMEM100 was significantly reduced in the two groups, so we hypothesized a close relation of TMEM100 in the generation or regulation of pain. Interestingly, although the expression of TMEM100 was significantly decreased in both CCI and TNI groups compared to the normal group, the decrease in TMEM100 was more pronounced in the CCI model. Many studies have demonstrated that [9] the downregulation of TMEM100 may be related to the proliferation of astrocytes and microglia after nerve injury. By detecting astrocyte-specific marker (GFAP) and microglia-specific marker (Iba-1), we discovered that there were different degrees of elevation in both pain models; in the CCI model, the elevation of GFAP and Iba-1 was more pronounced than that of TNI, which explained the decrease of TMEM100 in NP, and lower expression of TMEM100 in CCI compared to TNI model.

The function of TMEM100 is implicated in many aspects of biology. For example, TMEM100 is involved in the control of developmental proliferation and differentiation [40]. It plays a role in cell development and differentiation through pathways such as transforming growth factor-bone morphogenic protein in the enteric nervous system. It has essential functions in maintaining vascular integrity as well as in the formation of blood vessels. Meanwhile, TMEM100 acts as a tumor suppressor in various tumor cells to inhibit metastasis and proliferation [41]. Pan et al. [24] demonstrated that TMEM100 is crucial for the secretion of inflammatory factors and found that TNF-*α* had an inhibitory effect on the expression of TMEM100, while decreased TMEM100 expression could significantly reduce the secretion of inflammatory factors such as IL-1*β* and IL-6. This is consistent with the findings of our study that the expression of TNF-*α*, IL-1*β*, and IL-6 in DRG of CCI and TNI rats decreased after overexpression of TMEM100. The release of inflammatory mediators (TNF-*α*, IL-1*β*, and IL-6) is closely related to the pathogenesis of NP. These inflammatory mediators contribute to central spinal cord sensitization, thereby enhancing the development of NP [42, 43].

Activation of glial cells and interactions between these cells and neurons may be involved in nociception in the central and peripheral nervous systems [44]. Glial cells, including astrocytes and microglia, are involved in the induction and maintenance of NP [45]. The vital role astrocytes play upon activation may be related to the production of cytokines after injury [46]. It has been suggested that upregulation of GFAP, a marker of astrocyte activation following injury, has a role in the maintenance of NP [47]. One study found that upregulation of GFAP persisted from 3 to 21 days after nerve injury [48]. This study showed that GFAP levels increased in the groups of CCI and TNI models injected with empty virus (AAV6-GFP group). In contrast, the group injected with a virus carrying TMEM100 (AAV6-TMEM100) exhibited attenuated GFAP levels in CCI and TNI models.

Furthermore, the activation of microglia has a key role in the central sensitization of NP [49]. The pathological condition of NP results in microglia activation: microglia release many pro-inflammatory cytokines, such as TNF-*α*, along with glutamate release, excess reactive oxygen, and apoptosis. In this study, we detected different degrees of elevation of Iba-1, a marker of microglia activation, among the rats of AAV6-GFP group in the CCI and TNI models. The levels of Iba-1 in the AAV6-TMEM100 group were significantly decreased. Yu et al. [9] proved through in vitro experiments that overexpression of TMEM100 in astrocytes and microglia cell lines significantly inhibited their proliferation and found through animal experiments that TMEM100 may play a role in the control of satellite glial cells (SGCs) proliferation. It is believed that glial cell proliferation in animals after nerve injury may be the reason for the downregulation of TMEM100 expression, which is consistent with our findings.

After we injected AAV6-TMEM100 into SN of rats, the expression of TMEM100 in both CCI and TNI rats was significantly increased, and the expression of pain behavior was significantly improved, which also reflected the potential therapeutic mechanism of analgesic effect of TMEM100. Therefore, AAV6-mediated DRG-targeted delivery of TMEM100 has the potential to be translated into clinical use for treating patients with NP, although long-term safety requires further study.

## 5. Conclusions

This study found that the expression level of TMEM100 was decreased in NP. By upregulating the expression of TMEM100, the activation of glial cells and inflammatory mediators can be reduced to relieve pain. We believe that TMEM100 may be helpful in the treatment of NP.

## Figures and Tables

**Table 1:** The primers used for qPCR detection.

Gene name	Forward	Reverse
TMEM100	GTCTTCATCACCGGGATCGT	TGTTCCTTTGTCTCACCTTCCA
GAPHD	ATGCCGCCTGGAGAAACC	GCATCAAAGGTGGAAGAATGG
IL-1*β*	CCCAAGCACCTTCTTTTCCTT	TCAGACAGCACGAGGCATTT
IL-6	CTGATTGTATGAACAGCGATGATG	GGTAGAAACGGAACTCCAGAAGAC
TNF-*α*	CAAGAGCCCTTGCCCTAAGG	CGGACTCCGTGATGTCTAAGTACTT
GFAP	GAGATCGCCACCTACAGGAAATT	CTTTACCACGATGTTCCTCTTGAG
Iba1	GGAGGCCTTCAAGACGAAGTAC	GAGCCACTGGACACCTCTCTAATT

**Table 2:** Primary antibodies and IgG controls used in this study.

Antibody^*∗*^	Host	Supplier/catalog number	Dilution
TMEM100	Rabbit polyclonal	Millipore/ABN1721	1 : 100(IHC), 1 : 500(Wb)
TMEM100	Mouse monoclonal	Origene/TA500532	1 : 100(IHC), 1 : 500(Wb)
GFAP	Mouse monoclonal	Elabscience/E-AB-22022	1 : 200(IHC), 1 : 1000(Wb)
Iba1	Rabbit polyclonal	Abcam/ab178846	1 : 100(IHC), 1 : 1000(Wb)
IL-1*β*	Rabbit polyclonal	Affinity/AF5103	1 : 1000(Wb)
IL-6	Rabbit polyclonal	Affinity/DF6087	1 : 1000(Wb)
TNF-*α*	Rabbit polyclonal	Abcam/ab215188	1 : 1000(Wb)
*β*-actin	Rabbit polyclonal	Elabscience/E-AB-20058	1 : 1000(Wb)
Tubulin	Rabbit polyclonal	Elabscience/E-AB-20070	1 : 200(IHC)
IgG control	Mouse	Elabscience/E-AB-1001	1 : 2000(Wb)
IgG control	Rabbit	Elabscience/E-AB-1003	1 : 5000(Wb)
IgG control	Mouse	Elabscience/E-AB-1015	1 : 100(IHC)
IgG control	Rabbit	Elabscience/E-AB-1014	1 : 100(IHC)

^*∗*^TMEM100, transmembraneprotein100; GFA*P*, glial fibrillary acidic protein; Iba1, ionized calcium binding adaptor molecule 1; IL-1*β*, interleukin-1*β*; IL-6, interleukin-6; TNF-*α*, tumor necrosis factor *α*; GAPDH, glyceraldehyde 3-phosphate dehydrogenase; Tubulin, *β*-tubulin; IgG, immunoglobulin G.

## Data Availability

All the data and material can be available from Zhaoyang Guo, Zhu Guo, Zuoran Fan, Hongfei Xiang, Xiaolin Wu, and Bohua Chen for reasonable request.
